# The Multifaceted Roles of Neutrophil Death in COPD and Lung Cancer

**DOI:** 10.70322/jrbtm.2024.10022

**Published:** 2024-12-09

**Authors:** Arabella Wan, Dongshi Chen

**Affiliations:** 1 Division of Pulmonary, Critical Care and Sleep Medicine, Department of Medicine, Keck School of Medicine, University of Southern California, Los Angeles, CA 90033, USA; 2 Hastings Center for Pulmonary Research, Keck School of Medicine, University of Southern California, Los Angeles, CA 90033, USA

**Keywords:** Nuetrophil, Cell death, COPD, Lung cancer

## Abstract

Chronic obstructive pulmonary disease (COPD) and lung cancer are closely linked, with individuals suffering from COPD at a significantly higher risk of developing lung cancer. The mechanisms driving this increased risk are multifaceted, involving genomic instability, immune dysregulation, and alterations in the lung environment. Neutrophils, the most abundant myeloid cells in human blood, have emerged as critical regulators of inflammation in both COPD and lung cancer. Despite their short lifespan, neutrophils contribute to disease progression through various forms of programmed cell death, including apoptosis, necroptosis, ferroptosis, pyroptosis, and NETosis, a form of neutrophil death with neutrophil extracellular traps (NETs) formation. These distinct death pathways affect inflammatory responses, tissue remodeling, and disease progression in COPD and lung cancer. This review provides an in-depth exploration of the mechanisms regulating neutrophil death, the interplay between various cell death pathways, and their influence on disease progression. Additionally, we highlight emerging therapeutic approaches aimed at targeting neutrophil death pathways, presenting promising new interventions to enhance treatment outcomes in COPD and lung cancer.

## 1. Introduction

Chronic obstructive pulmonary disease (COPD) and lung cancer are two leading causes of morbidity and mortality worldwide [1–3]. Although COPD and lung cancer share common risk factors, including tobacco smoking, air pollution, and occupational exposure to metals, dust, and fumes [4], the relationship between these two diseases is complicated. Lung cancer significantly contributes to the increased morbidity and mortality observed in COPD patients [5]. Reversely, the presence of COPD is associated with reduced overall survival in lung cancer patients, particularly those with emphysematous predominant components [6]. Notably, squamous cell carcinoma (SCC), a subtype of non-small cell lung cancer (NSCLC), is more closely associated with COPD, showing a higher risk and decreased overall survival in affected individuals. Furthermore, the presence of COPD independently increases the risk of small cell lung cancer (SCLC), even in non-smokers [7,8].

A key link between COPD and lung cancer is chronic inflammation, a prevalent feature in both diseases [4]. Persistent inflammation in the lung tissues of COPD patients has been shown to increase the risk of lung cancer development by two to seven folds, irrespective of smoking history [9]. Dysregulation of the lung immune microenvironment plays a critical role in forming a tumor-promoting niche in both COPD and lung cancer [2,10]. Neutrophils, the most abundant circulating leukocytes in humans, are central to host defense and the acute phase responses [11]. However, in COPD, neutrophil inflammation is associated with increased disease severity and frequent exacerbations, while in lung cancer, a high neutrophil-to-lymphocyte ratio (NLR) correlates with poor prognosis and survival [12].

Historically, neutrophils were viewed as poorly adaptive cells with a short lifespan and limited effector functions, primarily involved in immediate immune responses [13]. This hypothesis has been challenged by recent discoveries, which highlight the plasticity of neutrophils under various conditions, including during inflammatory processes and cancer [14,15]. As critical immune cells, neutrophils show diverse functions depending on the disease context, driven by their plasticity, lifespan, and specific stimuli [16,17]. While previous studies focused primarily on the functions of living neutrophils and their subtypes in cancer and other inflammatory lung diseases [17,18], recent findings suggest that a deeper understanding of neutrophil death mechanisms is crucial to fully explain their diverse roles in pathological conditions [16,19].

This review focuses on the mechanisms of neutrophil death and their fundamental impact on COPD and lung cancer. By discussing the distinct forms of neutrophil death—apoptosis, necroptosis, ferroptosis, pyroptosis, and NETosis—we aim to provide a comprehensive understanding of how these processes influence disease outcomes and highlight potential therapeutic targets for these interconnected conditions.

## 2. Neutrophil Homeostasis and Death

Neutrophils, as the predominant type of leukocytes in human blood, serve as critical responders in the first line of defense against pathogens [20,21]. When inflammation or infection occurs, neutrophils rapidly mobilize from the bone marrow, traverse the sinusoidal endothelium, and eventually squeeze into the blood vessels [22]. This mobilization is tightly regulated by key factors, including C-X-C motif chemokine receptor 2 (CXCR2), CXCR2 ligands, and granulocyte colony-stimulating factor (G-CSF), in conjunction with CXCR4 and CXCR4 ligands [23]. Once their function in immune defense is completed, neutrophils undergo apoptosis and are typically cleared by macrophages in the liver, spleen, and bone marrow [24,25]. Alternatively, macrophages can phagocytose apoptotic neutrophils directly at sites of inflammation, ensuring their removal [26].

The controlled process of neutrophil death and subsequent clearance is essential for keeping the immune balance and ensuring the effective resolution of inflammation. Usually, neutrophils at inflammation sites undergo spontaneous apoptosis [27], a process facilitated by death receptor ligands, including Fas-ligand and tumor necrosis factor-*α* (TNF-*α*) released by infiltrating macrophages [28]. Furthermore, the engulfment of specific bacteria by neutrophils can accelerate their apoptosis at infection sites, thereby aiding the safe disposal of engulfed pathogens and the subsequent driving production of anti-inflammatory cytokines by macrophages [24,29]. This interplay promotes the resolution of inflammation and prevents excessive tissue damage.

**Figure 1.**
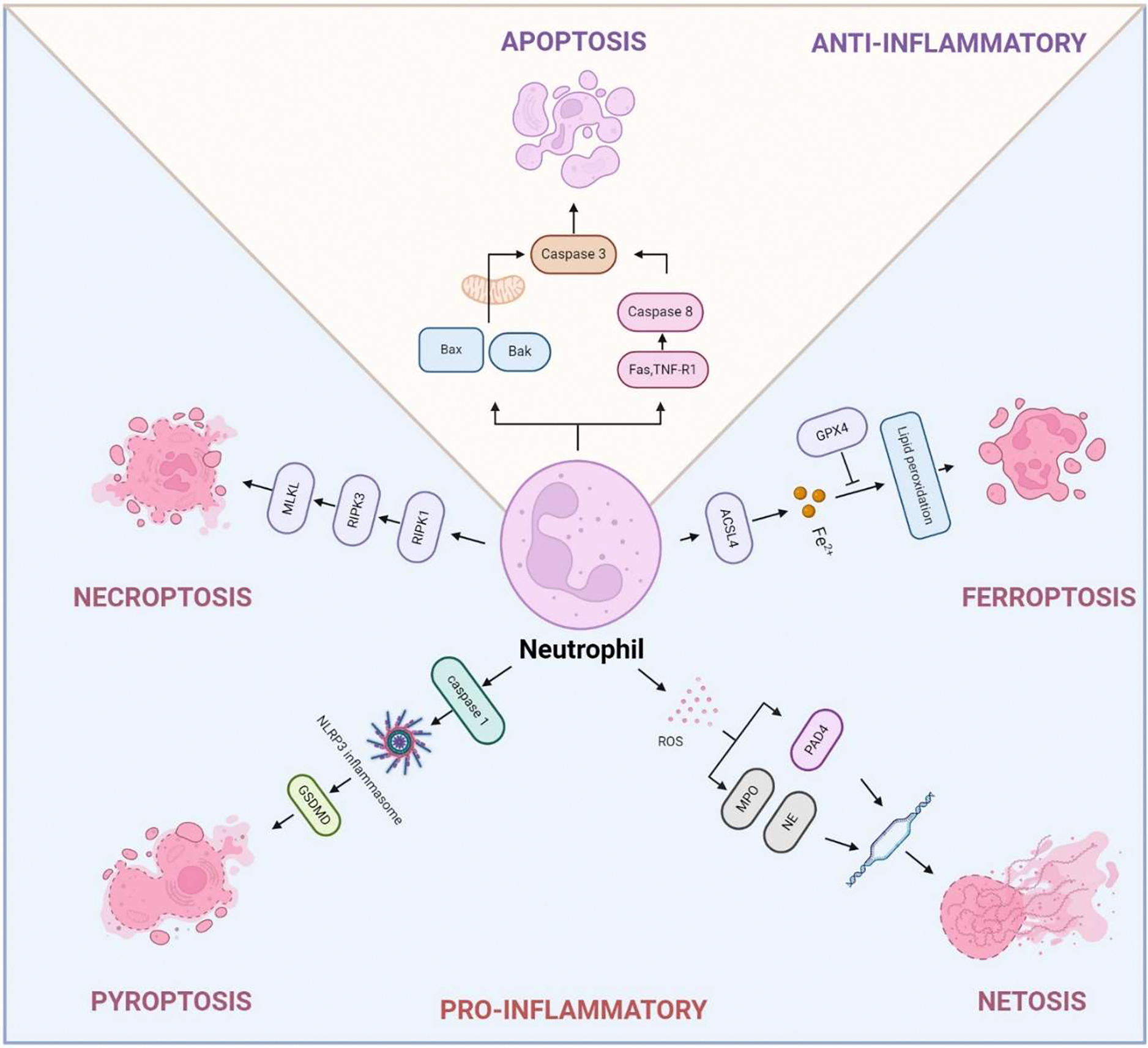
Schematic diagram of neutrophil death pathways. This figure illustrates the different pathways of neutrophil death, including apoptosis, necroptosis, pyroptosis, ferroptosis, and NETosis. It also shows key molecular players in each pathway, such as caspases in apoptosis, RIPK1/RIPK3 in necroptosis, GSDMD in pyroptosis, iron-dependent lipid peroxidation in ferroptosis, and the formation of neutrophil extracellular traps (NETs) in NETosis.

However, dysregulation of neutrophil death is often linked to chronic inflammatory diseases. Prolonged survival of neutrophils due to inhibition of apoptosis contributes to their accumulation at inflammatory sites, resulting in neutrophilia, a condition often observed in infectious and autoimmune diseases [30]. Abnormal dead neutrophils can exacerbate inflammation and cause tissue damage in COPD and lung cancer due to the excessive release of NETs, damage-associated molecular patterns (DAMPs), cytokines, chemokines, and toxic metabolites [26,31,32]. Therefore, understanding the molecular mechanisms that govern neutrophil death under both physiological and pathological conditions is essential for the development of therapeutic strategies to modulate inflammation and prevent tissue injury (Figure 1).

## 3. Ways of Neutrophilic Cell Death

Mature neutrophils, characterized by their lobulated nuclei and short lifespan of less than 24 h, undergo various forms of cell death, each with distinct consequences for health and disease [33]. Every day, billions of neutrophils die in the human body, primarily through apoptosis, but also via other mechanisms such as pyroptosis, necroptosis, ferroptosis, and NETosis [31,32]. Each pathway plays a unique role in the immune response and the pathogenesis of diseases like COPD and lung cancer (Table 1).

### 3.1. Apoptosis

Apoptosis represents the primary mode of neutrophil death, playing a critical role in maintaining neutrophil homeostasis and regulating inflammation. Similar to other cell types, the apoptosis of mature neutrophils is regulated by both intrinsic and extrinsic apoptotic signaling pathway [31]. In the intrinsic pathway, Bcl-2 family proteins, specifically Bax and Bak, form dimers in the mitochondrial outer membrane, causing mitochondrial outer membrane permeabilization (MOMP). This process triggers the release of cytochrome c into the cytoplasm, which initiates the activation of caspases, the central effectors of apoptosis [34]. In contrast, the extrinsic pathway is triggered by the activation of cell surface death receptors, including TNF-receptor 1 (R1) (TNFR1), FAS, and TRAIL receptors, which belong to the TNF receptor superfamily. This interaction activates Caspase-8, which facilitates MOMP, and subsequently activates Caspase-3, the key executor of apoptosis [35]. Apoptotic neutrophils are cleaned by macrophages, a process essential for the efficient resolution of inflammation.

### 3.2. Pyroptosis

Pyroptosis is an inflammatory form of cell death primarily observed in macrophages and dendritic cells, though it can also occur in neutrophils under certain conditions [36]. In contrast to apoptosis, a non-inflammatory process where vesicles remain intact with encapsulated cellular contents, pyroptosis is a specialized form of lytic cell death inherently linked to inflammation [37]. In response to exogenous pathogens (e.g., lipopolysaccharides (LPS), bacteria, viruses) or endogenous signals (e.g., cellular damage or stress-induced release of DAMPs like nucleic acids, uric acid, and ATP), immune cells such as neutrophils and macrophages rely on Pattern Recognition Receptors (PRRs) to detect these molecular patterns, initiating inflammasome assembly. This activation typically involves canonical complexes, including Absent in melanoma 2 (AIM2) or nucleotide-binding domain and leucine-rich repeat receptor (NLR), the adaptor protein ASC, and the effector molecule Caspase-1 [31]. During pyroptosis, caspase-1 activated by the inflammasome cleaves gasdermin D (GSDMD), which disrupts the cell membrane, leading to cell swelling, lysis, and release of cytoplasmic content [38]. Recent studies have shown that non-canonical inflammasome activation can also elicit GSDMD-dependent pyroptosis via caspase-11 activation [36]. Pyroptosis in neutrophils contributes to eliminating pathogens, but excessive pyroptosis can exacerbate tissue damage and promote lung inflammation [39].

### 3.3. Necroptosis

Neutrophil death by necroptosis usually acts as a trigger for inflammation [32]. This cell death is regulated by RIPK1, RIPK3, and MLKL, ultimately leading to disrupted membrane integrity and the release of cytosolic contents [40]. Unlike apoptosis, necroptosis lacks the characteristic morphological changes and does not involve DNA fragmentation [41]. Various stimuli, including activation of cell death receptors such as TNFR1, engagement of TLRs, IFNGR, adhesion receptors like CD11b, CD15, or CD18, monosodium urate crystals, and phagocytosis of Staphylococcus aureus, have been identified to trigger pathways that activate the RIP3-MLKL complex [41,42]. The upstream signaling leading to RIP3-MLKL activation varies depending on the receptors involved [41]. The necroptotic neutrophils have a rapid membrane rupture and release a large number of cellular contents, including cytokines, DAMPs, et al., which will lead to extensive tissue injury and inflammation [31]. Therefore, understanding the mechanism of neutrophil necroptosis will help elucidate the different functional roles of neutrophils in COPD and lung cancer.

### 3.4. Ferroptosis

Ferroptosis represents a specialized mechanism of programmed cell death driven by iron-dependent oxidative stress. It is characterized by the excessive peroxidation of phospholipids containing polyunsaturated fatty acid in cell membranes, leading to lethal levels of oxidative damage [43]. Unlike other forms of cell death, the buildup of lipid peroxides compromises membrane integrity, leading to cell death characterized by the loss of membrane structure and function, ultimately culminating in ferroptotic cell death [44]. Ferroptosis is majorly orchestrated by the interaction of three primary pathways: the GSH/GPX4 pathway, the NAD(P)H/FSP1/CoQ10 modulated lipid peroxidation, and the iron metabolism pathway [31]. Although research on ferroptosis has expanded rapidly, its effects on neutrophils are still not fully understood. Neutrophils undergoing ferroptosis mainly display mitochondrial dysfunction [45], along with mild morphological alterations and elevated levels of ROS and lipid peroxides (Figure 1, Table 1). Ferroptosis in neutrophils has been linked to a range of pathological conditions, such as inflammation, infection, and cancer [43,45,46]. Understanding the mechanisms of ferroptosis in neutrophils could provide insights into therapeutic strategies for diseases where neutrophil activity and cell death contribute to pathology.

### 3.5. NETosis

Identified in 2004, NETosis is a distinct type of neutrophil cell death characterized by the release of neutrophil extracellular traps (NETs). These traps consist of DNA strands coated with neutrophil-derived proteins, specifically structured to trap and neutralize invading pathogens [47]. In contrast to apoptosis, neutrophils undergoing NETosis exhibit disintegration of the nuclear and cellular membrane. However, their cytoplasmic contents remain intact for a brief period, allowing antimicrobial granule proteins to interact with the DNA [48,49]. The release of decondensed DNA during NETosis forms NETs that entrap microorganisms, along with highly toxic associated histones. The release of NETs is often paired with the secretion of neutrophilic enzymes like myeloperoxidase (MPO) and neutrophil elastase (NE), both of which play key roles in pathogen elimination [48]. While NET formation was initially recognized as a distinctive form of neutrophil cell death known as NETosis, later research revealed that NETs can also be produced independently of cell death [50].

NETosis can be triggered by a wide range of stimuli, including pathogens, lipopolysaccharides (LPS), antibodies, immune complexes, cytokines like TNF-*α* and IL-8, complement C3, microcrystals, cholesterol crystals, and certain drugs, including phorbol myristate acetate (PMA), calcium ionophores, and potassium ionophores [31,32,48]. NETosis is regulated by peptidyl arginine deaminase 4 (PAD4), an enzyme with a nuclear localization sequence that resides principally in the cytosol of resting neutrophils but can migrate to the nucleus, where it citrullinates histones and transcription factors [29]. By catalyzing the deamination of arginine on histone H3, PAD4 disrupts heterochromatin protein HP1B binding near lysine 9, causing chromatin de-condensation. The decondensation is further amplified by NE and MPO, enzymes housed in azurophilic granules and released in response to ROS generation within the cell. During NETosis, NE translocates to the nucleus, where it cleaves histones, facilitating chromatin decondensation [51]. These synchronized actions ultimately result in the rupture of nuclear and cellular membranes, allowing the release of NETs [52]. While the release of NETs is crucial for trapping and neutralizing pathogens, excessive NETs release will cause tissue damage, amplify inflammation, and modulate the immune response directly, which is closely correlated with COPD and lung cancer.

### 3.6. Crosstalk of Cell Death Pathway

**Figure 2.**
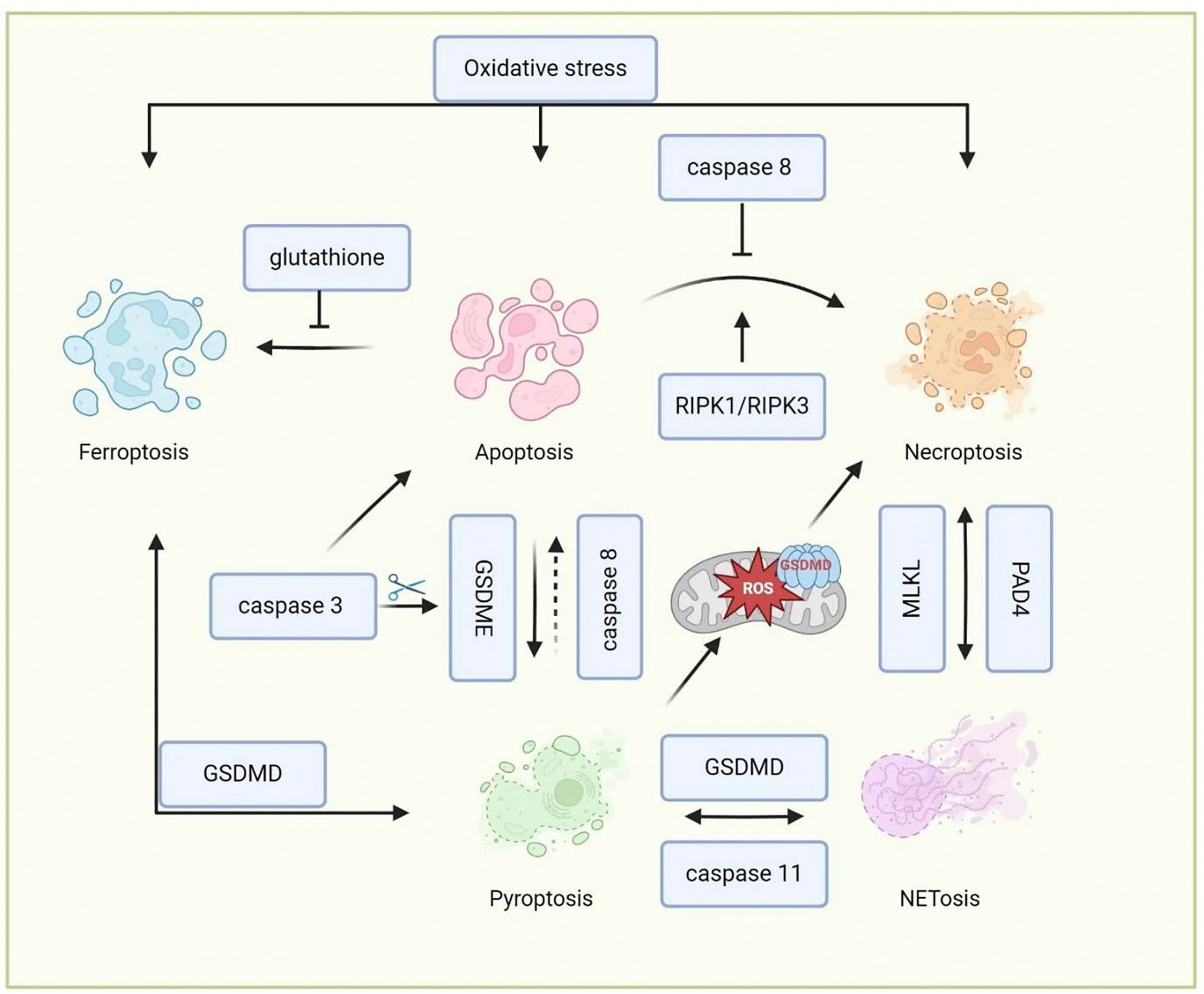
Crosstalk between neutrophil death pathways. This figure illustrates the crosstalk between various neutrophil death pathways, emphasizing how signaling interactions influence cell fate. Inhibition of caspase-8 shifts apoptosis to necroptosis via RIPK1/RIPK3, while shared signals like TNF receptor activation link both pathways. Caspase-3 activation can lead to either apoptosis or pyroptosis by cleaving GSDME. The diagram also highlights how oxidative stress in ferroptosis can amplify apoptotic signals and create feedback loops. Additionally, the release of DAMPs from necroptosis, pyroptosis, and ferroptosis amplifies local inflammation and can trigger further cell death pathways, with the NETs formation being influenced by GSDMD and MLKL.

The crosstalk between different cell death pathways in neutrophils is complicated, involving a network of signaling interactions that can influence the mode of cell death and its outcomes (Figure 2 and Table 2). In some cases, neutrophils that are defective in undergoing apoptosis may undergo alternative forms of cell death. Inhibition of caspase-8 can lead to the activation of RIPK1 and RIPK3, shifting the cell death pathway from apoptosis to necroptosis [53]. This switch is often regulated by the availability of caspase inhibitors and the presence of pro-inflammatory cytokines like TNF-*α*. Both apoptosis and necroptosis share upstream signals, including TNF receptor activation [54]. While caspase-3 and - 7 are primarily involved in apoptosis, the cleavage of gasdermin E (GSDME) by caspase-3 activation also leads to pyroptosis [55]. Additionally, the activation of inflammasomes, which cleaves GSDMD to promote pyroptosis, can prime cells for apoptosis through caspase-8 under certain contexts [55]. Recent studies have shown that PAD4 also mediates NET formation in apoptotic neutrophils upon disruption by GSDME [56], indicating the interplay of NETosis, pyroptosis, and apoptosis in neutrophils. Oxidative stress, a key player in ferroptosis, can also influence apoptotic pathways [57]. For example, the depletion of antioxidants like glutathione can drive the balance toward ferroptosis in cells that are primed for apoptosis [58]. The release of ROS and lipid peroxides during ferroptosis can amplify apoptotic signals to aggravate cell death.

Beyond apoptosis, other forms of cell death, such as necroptosis, pyroptosis, and ferroptosis, result in the release of DAMPs. These DAMPs can amplify local inflammation and influence the activation of other cell death pathways through autocrine and paracrine signaling loops [31,32]. There is considerable overlap in the signaling pathways involved in these different forms of cell death. For example, the lipid peroxidation seen in ferroptosis can also occur in necroptotic cells due to ROS production, creating a feedback loop that further exacerbates cell death [59]. The GSDMD pores formed during pyroptosis can increase membrane permeability, facilitating iron influx and lipid peroxidation, thereby linking pyroptosis to ferroptosis [59]. The GSDMD pores formation in the mitochondria also leads to mitochondria ROS release, which promotes a switch from pyroptosis into necroptosis [60]. Additionally, these various forms of cell death can also lead to NET formation and the release of neutrophil proteinases [38,45,61–63], which may function similarly to NETosis. For example, Chen et al. demonstrated that neutrophils undergo NETsosis in response to LPS or cytosolic Gram-negative bacteria, which relies on the cleavage of GSDMD by caspase-11 [36]. D’Cruz further demonstrated that the necroptotic cell death effector MLKL can activate PAD4-dependent NET formation upon bacterial infection [61]. However, the precise interconnections between these signaling pathways in neutrophils remain unclear and need further investigation. Recent studies showed the metabolism changes of neutrophils can determine the cell death signal of neutrophils upon different cellular stress [64,65]. Future studies may focus on investigating the neutrophil metabolic changes and their function in determining the cellular signaling of neutrophil death.

## 4. Neutrophil Cell Death in COPD and Lung Cancer

Neutrophil homeostasis involves a delicate equilibrium among processes such as maturation, aging, migration, and programmed cell death. The specific pathway of neutrophil death is determined by their interactions with microbial agents and external environmental stimuli, each leading to distinct physiological and immunological outcomes.

### 4.1. Neutrophil Cell Death in COPD

**Figure 3.**
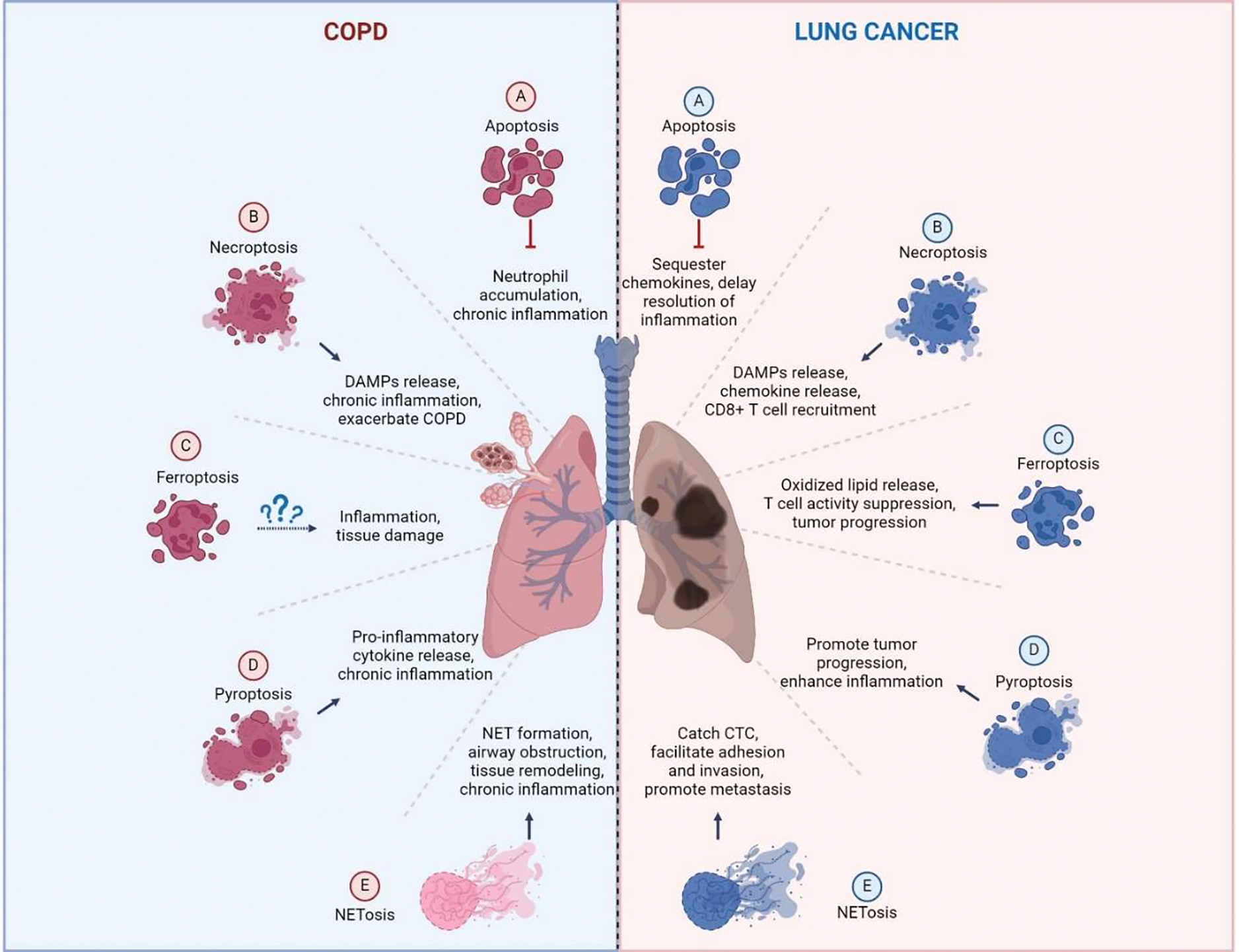
Interaction between neutrophil death pathways and disease progression in COPD and lung cancer. This figure demonstrates how the different neutrophil death pathways contribute to the pathogenesis of COPD and lung cancer. The left panel illustrates the role of apoptosis in resolving inflammation, necroptosis and pyroptosis in exacerbating chronic inflammation, and NETosis in promoting tissue remodeling in COPD. In lung cancer, the right panel shows how abnormal neutrophil death supports tumor growth, angiogenesis, and metastasis.

Neutrophils are key cellular mediators in the pathophysiology of COPD, irrespective of the clinical phenotype (such as chronic bronchitis, emphysema, or eosinophilic COPD), the severity of the disease, the rate of progression, or the age at onset. The high number of neutrophils recruited to the lungs in COPD patients is known to positively correlate with sputum thickness [66], contribute significantly to the poor response to corticosteroid treatment, and associate with a worse prognosis. While previous studies have focused on neutrophil migration, production of reactive oxygen species, degranulation, phagocytosis, NETs formation, and the release of neutrophil proteinases in COPD [67], much less is known about the mechanisms of neutrophil death in this disease. Neutrophils can die through various pathways in COPD, including apoptosis, necroptosis, pyroptosis, and NETosis, each contributing differently to the disease process (Figure 3 and Table 3).

Apoptotic neutrophils are typically cleared by phagocytes through efferocytosis, a specialized form of stimulated micropinocytosis that differs from the traditional phagocytosis of microbial pathogens [68]. Neutrophil apoptosis is generally thought to prevent or restrict inflammation [69]. However, under pathological conditions such as inflammation or cancer, the lifespan of neutrophils can vary significantly, ranging from shorter to extended durations [70,71]. Studies have found a marked decrease in the proportion of sputum neutrophils undergoing spontaneous apoptosis in individuals with COPD. During acute COPD exacerbations, neutrophil granulocytes exhibit reduced spontaneous apoptosis [72]. Efficient removal of apoptotic cells through efferocytosis is essential to avoid secondary necrosis and the release of harmful pro-inflammatory substances, which can exacerbate inflammation and lead to additional tissue damage [73]. As such, inducing neutrophil apoptosis may thus be a process that limits tissue injury and helps resolve inflammation, potentially slowing the progression of COPD.

Another critical form of neutrophil death in COPD is NETosis, which is associated with NET formation and the release of neutrophil proteinases. NETs formation by NETosis leads to higher extracellular DNA release, which was found in COPD patients, and correlated with absolute neutrophil counts in sputum and the degree of airway obstruction [74]. These results indicate that NETosis activity is significantly heightened in the airways of patients with stable COPD. The inability to efficiently clear NETosis may lead to persistent neutrophilic airway inflammation in COPD [74]. Excessive NET production, along with the release of neutrophil proteinases, can exacerbate lung inflammation, contribute to airway obstruction, and promote tissue injury in COPD patients [75]. Mechanically, neutrophils from COPD patients release greater amounts of NETs, which is attributed to dysfunction in the mitochondrial respiratory chain [76]. The NETs released by NETotic neutrophils activate NF-κB-dependent autoimmunity through the cGAS/TLR9 signal pathway, which promotes long-term CS exposure-induced COPD in preclinical mouse models [76]. However, the exact pathophysiological role of NETs in driving airway inflammation and lung injury in COPD patients remains incompletely understood.

Besides NETosis, other neutrophil death pathways, including necroptosis [61], pyroptosis [62], and ferroptosis [45] also contribute to driving the NET formation and releasing neutrophil proteases. Unlike apoptosis, which typically suppresses inflammation, these forms of cell death often lead to the extensive release of DAMPs from disintegrating membranes, depending on the cellular environment [37,44,77]. Therefore, we believe that pyroptotic, necroptotic, or ferroptotic neutrophils may contribute to the pathogenesis of COPD by driving chronic inflammation, causing tissue damage, and disrupting normal repair mechanisms. However, their exact roles in COPD still need further investigation.

### 4.2. Neutrophil Cell Death in Lung Cancer

Neutrophils, being among the most prevalent leukocytes in the immune system, respond to the tumor microenvironment (TME) and can polarize into distinct phenotypes: anti-tumorigenic (N1) or pro-tumorigenic (N2) [78]. Recent studies revealed that different types of neutrophil death can also significantly influence chronic inflammation and cancer progression. The type of cell death neutrophils undergo—whether apoptosis, necroptosis, ferroptosis, pyroptosis or NETosis—can have varying effects on cancer progression (Figure 3 and Table 3).

Neutrophil apoptosis, a form of programmed cell death, is crucial for resolving inflammation and maintaining tissue homeostasis. Effective clearance of apoptotic neutrophils by macrophages (efferocytosis) is essential for resolving inflammation. However, dysregulation of neutrophil apoptosis can extend the lifespan of tumor-associated neutrophils (TAN) [79], which contributes to a pro-tumorigenic environment, potentially leading to lung cancer development [80–82]. The prolonged survival of TANs can result in the accumulation of aged CXCR4^+^ neutrophils, which will suppress the function of CD8^+^ T cells and be associated with poor clinical outcomes [79]. Long-lived neutrophils may also develop into aged neutrophils, which can suppress T cell activity through PD-L1 induction [83]. Furthermore, extended survival promotes the enhanced immunosuppressive function of CD8^+^ T cells by TANs, thereby facilitating the *in vitro* and *in vivo* progression and growth of human LSCC tumors. Apoptotic neutrophils have been reported to sequester chemokines in the TME through modulation of CCR5 expression, which helps to resolve the inflammation [81]. Tumors and their microenvironments can delay neutrophil apoptosis by inducing PD-L1 expression or suppressing IFN signaling to exploit their pro-angiogenic and pro-metastatic properties [80,82]. While inducing neutrophil apoptosis *in vivo* may seem beneficial, it must be approached cautiously, as it may cause more harm than good if clearance mechanisms are not simultaneously enhanced.

Apoptosis-defective neutrophils can undergo NETosis, forming extracellular traps composed of expelled DNA strands and neutrophil proteinases. Neutrophils undergoing NETosis can eliminate pathogens through NETs release [47], and the secretion of neutrophil proteinases [84]. The components of NETs, including DNA, and proteinases, play a significant role in enhancing cancer cell proliferation, adhesion, migration, and invasion, thereby driving lung tumor growth and metastasis [85–87]. Research indicates that NETs aid metastatic tumor cells in spreading and colonizing host tissues by trapping circulating tumor cells (CTCs) and promoting angiogenesis [86,88]. NETs have been implicated in the progression and metastasis of NSCLC in several studies [85,89]. NETs can also promote lung tumor cell proliferation via NE secretion [90]. Moreover, the release of NETs also contributes to chemotherapy resistance via TGFβ activation in many cancer types, including lung cancer [91–93]. In this case, tumor cells may exploit NETosis to evade detection by immune cells and enhance tumor growth, invasion, and metastasis.

Ferroptosis, another form of neutrophil death, has recently been linked to tumor progression. The ferroptosis pathway was first identified in metastatic colorectal cancer liver metastases through single-cell transcriptome analysis [94]. Further studies by Kim et al. revealed that spontaneous ferroptosis of PMN-Myeloid-derived suppressor cells (MDSCs) within the TME stimulates the secretion of PGE2 and the release of oxidized phospholipids, enhancing their immunosuppressive effects by modulating the activity of CD8^+^ T cells and tumor-associated macrophages [46]. These ferroptotic neutrophils release PGE2 and oxidized lipids via upregulation of fatty acid transport protein 2 (FATP2) and ACSL4, limiting the activity of CD8 T cells [46,95]. Inhibiting FATP2 pharmacologically has been shown to neutralize the immunosuppressive functions of PMN-MDSCs, effectively suppressing tumor progression and sensitizing to ICI therapy. This highlights ferroptosis as a modifiable immunosuppressive mechanism in PMN-MDSCs within the TME, positioning it as a promising therapeutic target to combat tumor growth. Conversely, research indicates that tumor-infiltrating neutrophils exhibit resistance to ferroptosis [96]. Mechanistically, these neutrophils produce itaconate, a metabolite that inhibits ferroptosis and sustains their viability within the TME. Therefore, future research will focus on how to manipulate ferroptosis in tumor-infiltrated PMN-MDSCs to create a more favorable TME, presenting a potential therapeutic strategy to counteract tumor progression.

In terms of necroptosis, the release of chemokines and DAMPs from necroptotic neutrophils can recruit additional immune cells, including CD8^+^ T cells, macrophages, and other neutrophils, leading to the activation of anti-tumor immunity [97]. On the other hand, necroptosis in neutrophils results in plasma membrane permeability, the release of chromatin and DAMPs, and a phenomenon potentially linked to neutrophil NETosis. A recent study showed that a pro-tumoral TAN cluster characterized by with HMGB1 overexpression may suppress anti-tumoral immunity in NSCLC patients [98]. Neutrophil necroptosis influences the TME and immunity regulation, with its impact on immunity varying between tumor types. A necroptosis score can correlate with a favorable or risky prognostic factor for various cancers [99,100]. Therefore, the functional role of neutrophil necroptosis in lung cancer development still needs further investigation.

### 4.3. Crosstalk of Neutrophil Death in COPD and Lung Cancer

The risk of lung cancer in patients with COPD is two- to five- folds greater compared to smokers without COPD [3,4,6]. While both COPD and lung cancer involve neutrophil recruitment, activation, and delayed apoptosis, the functional outcomes of neutrophil death differ significantly between these conditions. In COPD, neutrophil death primarily contributes to chronic inflammation and tissue destruction by releasing the proteolytic enzymes and ROS. In contrast, in lung cancer, the release of NETs, immunosuppressive cytokines, and proteolytic enzymes by abnormally dying neutrophils supports tumor growth, angiogenesis, and metastasis.

The abnormal death of neutrophils, including NETosis, necroptosis, ferroptosis, and pyroptosis, leads to the release of NETs, DAMPs, and neutrophil proteinases, thereby exacerbating inflammation. NETs have been implicated in promoting metastasis, aiding tumor cell survival in circulation, and facilitating tumor invasion [85,101]. Additionally, NETs and intact neutrophils can capture tumor cells through Mac-1/ICAM-1 interactions, enhancing tumor cell adhesion and promoting metastasis [85]. Beyond NETs, NE, a crucial neutrophil proteinase, is a key protease that significantly contributes to the development of emphysema in COPD [102] and has demonstrated pro-tumorigenic effects across various cancers, driving primary tumor growth and secondary metastasis [90]. In lung cancer patients, NE levels are notably elevated and show a positive correlation with disease progression. NE activity in bronchoalveolar lavage fluid (BALF) and serum is up to five times higher in lung cancer patients compared to those with COPD, highlighting its variable expression across different conditions [103]. Conversely, a recent study indicated that NE secreted by apoptotic neutrophils could kill 35 different human or murine cancer cell lines across 11 tumor types [104], suggesting that enhanced NE secretion could be explored as an anti-tumor strategy. However, escalated NE levels might also lead to lung tissue damage[102], and worsening COPD.

Neutrophils can undergo different forms of cell death due to their critical role in the immune response and the need to adapt to various pathogenic and environmental challenges. Each form of cell death serves distinct physiological and pathological purposes, allowing neutrophils to efficiently respond to different stimuli and conditions. Different forms of neutrophil death, such as apoptosis, necrosis, necroptosis, pyroptosis, and NETosis, can indeed lead to the release of various molecules, including NETs and proteinases. These distinct mechanisms and outcomes can have varied functional implications in diseases such as COPD and lung cancer. Therefore, understanding these mechanisms is crucial for developing targeted therapies to modulate neutrophil activity and mitigate disease progression.

## 5. Therapeutic Strategies and Future Directions

### 5.1. Targeting Neutrophil Death Pathways in COPD

Current therapies for COPD mainly focus on alleviating symptoms, such as bronchodilation, and reducing mucus production [105], but often fail to address the underlying inflammatory processes driven by neutrophil activity. Neutrophil death pathways—particularly apoptosis, necroptosis, and NETosis—are central to the chronic inflammation and tissue destruction observed in COPD. Therefore, targeting these pathways presents a promising approach to modifying disease progression and improving patient outcomes.

Modulating neutrophil apoptosis could play a crucial role in resolving inflammation in COPD. Enhancing the clearance of apoptotic neutrophils through efferocytosis may prevent secondary necrosis and the subsequent release of pro-inflammatory mediators that contribute to tissue damage [73]. For example, azithromycin has been shown to enhance macrophage-mediated efferocytosis and increase the expression of mannose receptors, thereby reducing chronic inflammation in COPD patients [106]. Although statins have demonstrated potential in enhancing efferocytosis and reducing neutrophil-driven inflammation (as evidenced by reductions in C-reactive protein and IL-6) in clinical analyses, these benefits have not yet translated into clear clinical outcomes for COPD patients [107]. Additionally, a screening study of kinase inhibitors identified several compounds, including the ErbB inhibitors gefitinib, CP-724714, erbstatin, tyrphostin AG825, which can accelerate neutrophil apoptosis, indicating their potential as therapeutic agents in neutrophil-driven inflammatory diseases such as COPD [108]. However, translating these findings into clinical practice requires more research and clinical trials to assess safety and efficacy in human patients.

Inhibiting NETosis represents another strategy for mitigating COPD-related inflammation. Excessive NET formation contributes to airway obstruction, persistent inflammation, and tissue remodeling in COPD [75]. Therapeutic interventions aimed at reducing NET formation, such as PAD4 inhibitors or mitochondrial-targeted antioxidants like mitoTEMPO, have shown promise in preclinical models [76]. By reducing NET formation, these therapies could decrease airway obstruction and slow the progression of COPD. Additionally, the use of DNase I to degrade extracellular DNA in NETs has been explored as a means to reduce NET-associated tissue damage [109]. Another approach involves neutralizing NE through the use of protease inhibitors like SLPI (secretory leukocyte protease inhibitor), which has been shown to eliminate excess NETs [110], providing an additional avenue for therapeutic intervention in COPD [111]. In the future, clinical trials are needed to evaluate the safety and efficacy of these approaches in human patients.

Advances in research on cell death pathways are identifying novel therapeutic targets, providing new opportunities to improve COPD outcomes by reducing inflammation. Specific inhibitors of key regulators in these pathways, such as RIPK3 for necroptosis or GSDMD for pyroptosis, are currently being explored in preclinical studies. RIPK3 inhibitors could be particularly valuable in diseases where necroptosis plays a significant role, such as COPD and certain types of lung cancer. Blocking RIPK3 can reduce necroptosis-induced inflammation and tissue damage, potentially slowing disease progression [77,112,113]. Inhibition of necroptosis was reported to resolve the LPS-induced lung inflammation by switching the neutrophil death into apoptosis [114], further suggesting that inhibition of necroptosis can be a solution for COPD therapy. GSDMD inhibitors, on the other hand, could be used to modulate pyroptosis, reducing the inflammatory response in diseases where this pathway is dysregulated [36]. Inhibiting GSDMD could prevent excessive inflammatory damage associated with pyroptosis, potentially offering therapeutic benefits in conditions like COPD, where inflammation plays a central role in disease progression [115]. These preclinical studies indicated that targeting necroptosis or pyroptosis in neutrophils might be potent in reducing inflammation in COPD. Ongoing research is focused on developing more selective and potent inhibitors that target neutrophils, which will provide novel therapies for COPD.

### 5.2. Targeting Neutrophil Death Pathways in Lung Cancer

In lung cancer, targeting neutrophil death pathways offers a novel approach to enhancing anti-tumor immunity and reducing metastasis. Neutrophils in the TME can either promote or inhibit tumor progression, depending on their mode of death. Therefore, modulating these pathways could shift the balance towards anti-tumorigenic activities.

Inhibiting NETosis presents a promising strategy in lung cancer therapy, as NETs have been shown to promote tumor cell adhesion, migration, and metastasis [86,88]. NET can also be indirectly targeted by inhibiting enzymes such as MPO, NE, and PAD4 [116]. For example, inhibiting MPO chlorination reduces levels of hypochlorous acid (HOCI, the main oxidation product of MPO *in vivo*), which can induce apoptosis in A549 lung cancer cells [117]. Additionally, targeting MPO using photosensitizers conjugated with 5-hydroxytryptamine or the drug Zileuton has been shown to inhibit neutrophil-mediated lung metastasis [118]. Inhibition of NET with DNase or NE inhibitors has also been shown to abrogate cancer metastasis by reducing microvascular NET deposition and preventing the trapping of circulating lung carcinoma cells within DNA webs [88]. Combining NETosis inhibitors with immune checkpoint inhibitors or other cancer therapies could further enhance treatment outcomes by reducing metastasis and improving immune-mediated tumor clearance [119,120]. For example, PAD4 inhibitors in combination with anti-PD-1 therapy could reduce tumor metastasis and enhance the efficacy of immune checkpoint inhibition. This combination approach could synergistically enhance the immune response against tumors by diminishing the immunosuppressive effects of NETs and improving T-cell infiltration into tumors [119].

Targeting ferroptosis in neutrophils offers another novel approach to lung cancer therapy. Ferroptosis, which involves iron-dependent lipid peroxidation, can drive tumor growth when dysregulated [43]. Modulating ferroptosis in tumor-infiltrating neutrophils could suppress tumor growth by enhancing the neutrophil-mediated killing of tumor cells. The ferroptosis inhibitors, Ferrostatin and liproxstatin, can suppress neutrophils ferroptosis in TME, and enhance tumor suppression when combined with anti-PD1 immunotherapy [46]. Additionally, targeting GSDMD in the TME could reduce tumor-associated inflammation and improve the effectiveness of cancer immunotherapies [121]. On the other hand, necroptotic cells can release DAMPs that stimulate immune responses, which could help trigger anti-tumor immune responses by recruiting and activating other immune cells like CD8^+^ T cells and macrophages [122]. Therefore, stimulating neutrophil necroptosis might benefit cancer patients with ICI therapy.

The choice of therapeutic strategy targeting neutrophil death depends on the specific disease context and the role of neutrophils in the disease pathology. Future research should focus on identifying biomarkers that predict patient response to these therapies, enabling more personalized treatment approaches. For example, biomarkers indicating elevated levels of NET formation, necroptosis, or pyroptosis could help identify patients who would benefit most from targeted therapies [123]. Additionally, exploring combination treatments that target multiple neutrophil death pathways simultaneously could provide synergistic benefits, enhancing therapeutic efficacy while minimizing adverse effects [31]. For instance, combining RIPK3 inhibitors with ferroptosis inducers could reduce inflammation while promoting tumor cell death in lung cancer patients [124]. Preclinical studies and subsequent clinical trials will be essential to translate these findings into effective treatments.

## 6. Conclusions

Neutrophil death is a pivotal process in the pathogenesis of both COPD and lung cancer. The specific pathways through which neutrophils die—whether through apoptosis, necroptosis, pyroptosis, ferroptosis, or NETosis—can significantly influence the inflammatory response, tissue remodeling, and overall disease progression. In COPD, the dysregulated death of neutrophils contributes to chronic inflammation, airway obstruction, and progressive lung tissue destruction. In lung cancer, the mode of neutrophil death can either suppress or enhance tumor growth and metastasis, influenced by the interplay between pro-tumorigenic and anti-tumorigenic signals. Therefore, unraveling these mechanisms is essential for designing innovative therapeutic approaches to modulate neutrophil activity to benefit patient outcomes in both diseases.

The potential to target neutrophil death pathways as a therapeutic strategy offers a promising approach to treating both COPD and lung cancer. Modulating these pathways could allow for more precise control over inflammation, reducing tissue damage and slowing disease progression. For instance, enhancing neutrophil apoptosis and its effective clearance could help resolve chronic inflammation in COPD, while inhibiting pathways like necroptosis and NETosis could reduce the harmful effects of excessive inflammation and prevent further tissue injury. In lung cancer, strategies targeting neutrophil death pathways could enhance anti-tumor immunity, reduce metastasis, and improve the efficacy of existing cancer therapies. The development of therapies that specifically target these pathways could lead to significant advances in the management of these chronic diseases.

In the future, research may focus on several key areas to fully harness the therapeutic potential of targeting neutrophil death pathways. First, studies should aim to elucidate the intricate crosstalk between different neutrophil death pathways and how these interactions influence disease progression in COPD and lung cancer. Understanding these interactions will be essential for developing combination therapies that can modulate multiple pathways simultaneously for a more comprehensive therapeutic effect. Second, since neutrophils interact closely with other immune cells, altering neutrophil death pathways could have broader implications for immune system function in COPD and lung cancer patients. It is crucial to strike the right balance when orchestrating neutrophil death, ensuring that it bolsters anti-tumor immunity while avoiding excessive inflammation or tissue damage. Third, there is a need for clinical trials to explore the safety and efficacy of targeting neutrophil death pathways in patients with COPD and lung cancer. These trials should assess not only the direct effects on disease progression but also their potential to reduce complications and improve overall patient outcomes. Finally, interdisciplinary research integrating insights from immunology, oncology, and respiratory medicine will be essential for advancing the field. Collaborative efforts across these disciplines can help translate basic research findings into clinical applications, leading to the development of innovative therapies that address the complex interplay between neutrophil death and disease pathology in COPD and lung cancer. As our understanding of these processes deepens, the potential to improve patient outcomes through targeted therapies will increase, offering hope for more effective treatments in the future.

## Figures and Tables

**Table 1.** Summary of neutrophil death pathways and their key molecular players.

Pathway	Morphology	Key Molecular Players	Key Triggers	Outcome in Neutrophils
Apoptosis	Cell shrinkage, chromatin condensation, membrane blebbing	Caspases (Caspase-3, -7, -8), Bcl-2 family proteins (Bax, Bak), cytochrome c	TNF-*α*, Fas ligand	Controlled cell death, Reduced inflammation
Necroptosis	Cell swelling, membrane rupture, release of DAMPs	RIPK1, RIPK3, MLKL	Caspase-8 inhibition, TNF receptor activation	Increased inflammation
Pyroptosis	Cell swelling, lysis, release of inflammatory cytokines	GSDMD, GSDME Caspase-1, Inflammasomes (NLRP3, AIM2)	Infection, DAMPs, PAMPs	Cell lysis, Amplified inflammatory response
Ferroptosis	Iron-mediated lipid peroxidation, loss of membrane integrity	Lipid peroxides, Iron, GPX4, ACSL4	Oxidative stress, Depleted antioxidants	Iron-dependent cell death, Amplified apoptotic signals
NETosis	Release of NETs (extracellular DNA traps), disintegration of nuclear membrane	Histones, NE, MPO, PAD4	Bacterial infection, Cytokines	Release of NETs, Tissue remodeling

**Table 2.** Neutrophil death pathways in COPD and lung cancer.

Pathway	Role in COPD	Role in Lung Cancer	Associated Outcomes
Apoptosis	Resolution of inflammation, Prevents chronic inflammation	Delayed apoptosis may promote tumor growth	Controlled inflammation in COPD, Tumor promotion in lung cancer
Necroptosis	Exacerbates chronic inflammation, Tissue damage	Promotes tumor progression, Immune suppression	Worsened COPD, Increased lung cancer metastasis
Pyroptosis	Amplifies inflammation, Contributes to exacerbations	Enhances inflammatory response, TME	Chronic airway inflammation in COPD, Tumor growth in lung cancer
Ferroptosis	Contributes to oxidative damage	May promote or inhibit tumor growth	Potential therapeutic target in lung cancer
NETosis	Leads to airway obstruction, Tissue remodeling	Facilitates metastasis, Promotes angiogenesis	Airway obstruction in COPD, Metastasis in lung cancer

**Table 3.** Therapeutic strategies targeting neutrophil death pathways in COPD and lung cancer.

Therapeutic Target	Mechanism of Action	Indication	Potential Therapeuitic Agents	Expected Outcome
Apoptosis Enhancers	Promotes efferocytosis, Clears apoptotic cells	COPD	Statins, CD31 modulators	Reduced chronic inflammation, slowed disease progression
Necroptosis Inhibitors	Inhibits RIPK1/RIPK3, Reduces inflammation	COPD, Lung Cancer	Necrostatins, RIPK inhibitors	Reduced necroptosis-induced inflammation
Pyroptosis Modulators	Inhibits inflammasome activation, Reduces cell lysis	COPD, Lung Cancer	GSDMD inhibitors	Reduce tumor-associated inflammation, and improve immunotherapie s
Ferroptosis Inducers/Inhibitors	Modulates ROS and lipid peroxidation, Targets iron metabolism	Lung Cancer	Ferroptosis inducers (e.g., Erastin, RSL3)	Enhanced tumor cell killing
NETosis Inhibitors	Inhibits PAD4, Degrades extracellular DNA	COPD, Lung Cancer	PAD4 inhibitors, DNase I	Reduced metastasis, enhanced immune response
